# Knowledge and Consequences of Violence Against Health Professionals in Southern Portugal

**DOI:** 10.3390/nursrep14040233

**Published:** 2024-10-27

**Authors:** Maria Otília Zangão, Laurência Gemito, Isaura Serra, Dulce Cruz, Maria da Luz Barros, Maria Antónia Chora, Carolina Santos, Anabela Coelho, Elisabete Alves

**Affiliations:** 1 CHRC—Comprehensive Health Research Centre, NOVA Medical School, University of Évora, 1150-082 Lisbon, Portugal; mlpg@uevora.pt (L.G.); iserra@uevora.pt (I.S.); dcruz@uevora.pt (D.C.); mlb@uevora.pt (M.d.L.B.); mafcc@uevora.pt (M.A.C.); anabela.coelho@uevora.pt (A.C.); elisabete.alves@uevora.pt (E.A.); 2 Nursing Department, São João de Deus School of Nursing, University of Évora, 7005-811 Évora, Portugal; 3 NOVA National School of Public Health, Public Health Research Centre, CHRC—Comprehensive Health Research Centre, NOVA University Lisbon, 1600-560 Lisbon, Portugal; c.santos@ensp.unl.pt; 4 Global Health and Tropical Medicine, Instituto de Higiene e Medicina Tropical, NOVA Medical School, 1099-085 Lisbon, Portugal

**Keywords:** workplace violence, violence, social behavior, health personnel, health knowledge, attitudes, practice, nursing

## Abstract

Background: Violence against health professionals is a global and growing problem, with significant impacts on the quality of care and the mental health of workers. Objectives: To analyze the level of knowledge, reporting practices and consequences of violence against health professionals in the Alentejo region (southern Portugal). Methods: This was an observational, cross-sectional study involving 440 health professionals (doctors and nurses). Data were collected using an online platform and a structured questionnaire created specifically for this study. In the statistical analysis, the data were described as counts and proportions and the X2 test was used considering a significance level of 0.05. Results: This study reveals that violence against health professionals in the southern region of Portugal is a frequent problem (40%), with a higher incidence among nurses (80%). Despite awareness of the existence and functioning of reporting channels, reporting is low (52%). The main causes are related to the health system, professionals and users. The consequences include mental health problems and a reduction in the quality of care provided. Suggested measures to combat violence include improving security, training and punishing aggressors. Conclusions: This study reveals that violence against health professionals in the southern region of Portugal is a frequent, under-reported problem with serious consequences for professionals and the quality of care.

## 1. Introduction

Violence against health professionals, a global problem that manifests itself in various ways, has been increasingly recognized as a public health and human rights issue [1,2,3]. The International Labour Organization (ILO) Convention on Violence and Harassment, 2019 (No. 190), recognizes the crucial importance of a comprehensive strategy that involves an inclusive, integrated and gender-sensitive approach in designing a response to the issue of workplace violence [4].

Violence against health professionals is not just an individual problem, but a social problem that affects everyone [5]. When you attack a health professional, you attack society as a whole, since you compromise the quality of care and devalue a profession that is essential to public health [6].

Understanding the magnitude and characteristics of violence against health professionals in the workplace is fundamental for the development of public policies and interventions that promote safer and healthier working environments [7]. By mapping the knowledge, opinions and experiences of health professionals, we hope to contribute to building a more humane and protected health system. There is no single approach to the problem of violence against health professionals in the workplace and a targeted response may require a more specific assessment of the context.

Knowledge about violence in the workplace is the first step towards preventing it. Many health professionals are not fully aware of their rights or the resources available to deal with situations of violence [8,9,10]. In 2020, the Action Plan for the Prevention of Violence in the Health Sector in Portugal was published [2]. One of the specific objectives of this plan is to learn about and investigate the phenomenon of violence in the health sector, leveraging the work previously carried out by the Observatory on Violence against Health Professionals, which was set up in 2006 and has demonstrated through its annual reports that it is a clear public health problem [11].

The lack of specific training on how to recognize and respond to signs of violence contributes to this situation of ignorance and underreporting. It is essential that professionals receive clear and practical guidance, from their initial training to the later stages of their careers, on the procedures to follow in the event of aggression. In addition, promoting awareness-raising campaigns in the workplace can help increase the level of knowledge on this crucial topic, which is one of the main international recommendations for reducing violence [2].

Reporting episodes of violence is a crucial factor in implementing protection measures and supporting the professionals affected [9,12]. The number of reports of violence against health professionals in Portugal fell by 37% in 2023 [13]. However, the low figures presented (1036 episodes of violence reported in 2023 vs. 1632 recorded in 2022) reveal a lack of safety culture when it comes to reporting these events. It is essential that all episodes are reported, as failure to do so makes intervention difficult [1]. Several factors contribute to this scenario, such as fear of retaliation, distrust in the effectiveness of reporting systems and lack of institutional support [14]. Many professionals choose not to report minor incidents, either because they do not think it will lead to significant changes or because they fear that the reporting process could make the situation even worse. This behavior creates a vicious circle, where failure to report perpetuates impunity and violence. In order to reverse this situation, it is necessary to create more accessible and confidential reporting mechanisms, as well as to ensure that reports result in concrete and effective action on the part of the competent authorities [9,10].

The consequences of violence against health professionals are wide-ranging and affect both the individuals directly involved and the health system as a whole [5,7,10]. Professionals exposed to situations of violence often face mental health problems such as anxiety, depression and burnout syndrome [15,16]. In addition, insecurity in the workplace can lead to a reduction in the quality of care provided, increased absenteeism and even professionals leaving the sector [15,16,17].

Violence also creates a climate of fear and mistrust among coworkers, which can compromise teamwork and therefore the effectiveness of health services. In the medium and long term, the lack of effective intervention can result in a crisis in the health sector, with a shortage of professionals and a deterioration in the services provided to the population [18,19], and increased direct and indirect costs for health systems [16].

In the Portuguese context, although there is a growing interest in tackling this issue, there are still few studies investigating the scale and characteristics of this phenomenon, particularly in the different regions of the country. The choice of the Alentejo region, located in the south of Portugal, as the focus of this research is justified by its socio-economic relevance and the diversity of work contexts in the health sector, in terms of rural and urban dimensions, the nature of the institutions or the professional areas. In addition, the region has demographic and cultural characteristics that can influence the occurrence and perception of violence in the workplace, as the World report on violence and health [5] highlighted that cultural specificity and tradition are sometimes presented as justifications for certain social practices that perpetuate violence. Identifying the risk factors associated with this problem helps to develop more effective prevention and mitigation strategies, in line with the recommendations of the World report on violence and health [5].

In a review of the literature, it was noted that the context and culture of the workplace also have significant impacts on the factors that contribute to workplace violence. The context is made up of a range of elements, including patient/visitor characteristics, healthcare worker characteristics, the physical environment and organizational, economic and socio-cultural factors [16].

The main objective of this study is to analyze the level of knowledge, reporting practices and consequences of violence against health professionals in the Alentejo region (southern Portugal).

## 2. Materials and Methods

This article describes an observational, cross-sectional study. The study was approved by the Ethics Committees of the University of Évora (Ref no. 22187) and the Local Health Units (LHUs) involved in the study. Free and informed consent was obtained from all participants in accordance with the guidelines of the ethics committees and bearing in mind the Declaration of Helsinki of the World Health Association.

Between January and May 2024, doctors and nurses working at the four LHUs in the Alentejo region (LHU Alentejo Central, EPE; LHU Alto Alentejo, EPE; LHU Baixo Alentejo, EPE; and LHU Litoral Alentejano, EPE) were invited to take part in the study via their institutional email. Of the 3525 health professionals invited to take part, 440 answered the questionnaire. We consider this sample to be representative of the population, with a confidence level of 95% and a margin of error of 5% (calculated using the calculator https://comentto.com/es/calculadora-muestral/ (Accessed on 16 August 2024).

LHUs are structures that integrate the care provided by health centers and hospitals, aiming at an integrated management model for the provision of health care to users, incorporating, legally and organizationally, hospital health care and primary health care in the same public entity [20].

Data were collected on an online platform using a structured questionnaire created specifically for the study. The questionnaire collected information on the sociodemographic characteristics of health professionals (gender, age, nationality and marital status), as well as work characteristics (professional category, place of work, type of contract, length of time working at the institution and working hours). The survey also covered aspects related to violence against health professionals in the workplace.

Information on the prevention of violence in the health sector was collected through questions aimed at finding out whether professionals were aware of the action plan for the prevention of violence in the health sector and the focal point of their institution, to whom they can turn for the clarification, exposure and reporting of any situation.

The “validation” of the questionnaire was carried out on a sample of 15 participants and consisted of legitimizing the content, clarity and comprehensibility, as well as semantic and cultural validation. We made sure that there was no need to make any changes and continued with the application. In the data analysis, we verified that the data were reliable and usable for analysis.

To collect data on the prevalence of violence at work, professionals were asked if they had ever been victims of violence at work and those who answered yes were directed to the questions where they reported the situation of violence. Data on the main reasons for not reporting the situation were determined using previously defined categories: fear of reprisals, lack of knowledge about how and where to report, complexity of the reporting process and distrust in the system and others (which should be specified). As for the main consequences of the episode of violence, several categories were presented, namely, no consequences, need for observation or minor treatment, need for medical procedure or treatment, need for prolonged treatment or prognosis of permanent damage, death, and do not know.

Data on the reactions of health professionals to an episode of violence were obtained through the open-ended question ‘How do you react to an incident of violence?’ The participants’ responses were then classified, through thematic analysis, into eight main categories: calm and measured reaction, emotional reaction (feeling angry, anxious, nervous or afraid), moving away from the aggressor, reporting to the authorities, devaluing the situation, reporting the incident at a higher level, seeking psychological support and seeking legal advice.

The procedures implemented in the health institution in response to the episode of violence were identified using a series of questions, including whether there was a need for any kind of treatment/monitoring, the need to take time off work, the need to submit a statement of accident at work to the superior, what steps were taken to investigate the causes of the episode, its possible prevention, the usual frequency with which these situations occur, the measures taken to support the victim, the evaluation of the support received, concern about violence in the workplace and satisfaction with the management of the incident.

Participants were asked to identify the three main factors that contribute to violence against health professionals, as well as the three most important measures to reduce such violence. The answers to these two open questions were analyzed using thematic analysis, with systematic coding of the data. Health professionals’ answers with similar meanings were inductively synthesized into categories and themes. Thus, three themes (characteristics of the health system, characteristics of health professionals and characteristics of users) and five themes (safety, organization/management, training/education, justice and user conditions) were defined, describing the most important factors that predispose to violence and the measures to reduce it, respectively.

Health professionals were also asked about the violence observed against other people in their workplace in the last 12 months. They were asked to indicate their gender, professional group (operational assistant, social worker, technical assistant, nurse, student/intern, pharmacist, doctor, emergency ambulance technician, diagnostic and therapeutic technician, senior health technician, psychologist, security guard, driver, doorman, does not know/answers and others), the relationship between the aggressor and the victim and the healthcare institution (patient/patient, patient/patient companion, visitors, group of citizens and healthcare professional from another institution/healthcare unit), the type of violence observed (physical, psychological, sexual, gender, racial/ethnic discrimination and others) and the consequences of the episode (none, need for observation or minor treatment, need for procedure or treatment, need for prolonged treatment or prognosis of permanent damage and do not know/answer).

Statistical analysis was carried out using IBM SPSS Statistics for Windows, version 29 (IBM Corporation, Armonk, NY, USA). Data were described as counts and proportions. Knowledge of violence prevention in the health sector, according to participants’ sociodemographic and work-related characteristics, was compared using the X2 test at a significance level of 0.05.

## 3. Results

The majority (74.8%) of the health professionals who took part in this study were female and more than half were aged between 34 and 55 (65.4%) (see Table 1). Almost all (96.4%) were Portuguese and almost 75% were married or living with a partner. The majority (80%) of the participants were nurses and two-thirds had indefinite employment contracts in public positions. The health professionals worked in the following LHUs: Alto Alentejo, EPE (51.6%), Alentejo Central, EPE (25.7%), Litoral Alentejano, EPE (16.6%) and Baixo Alentejo, EPE (6.1%). The majority (67.5%) of the participants had worked at the institution for 11 years or more and almost half (48.8%) worked shifts (Table 1).

Almost 60% of health professionals were aware of the existence of an action plan for the prevention of violence in the health sector and of the existence of a focal point in their institution to whom they can turn for clarification, exposure and notification of any situation (Table 2). Participants aged over 54 (*p* = 0.024 and *p* = 0.001), married or living together (*p* = 0.044 and *p* = 0.007) and working shifts (*p* < 0.001 and *p* = 0.021) were significantly more likely to be aware of the existence of an action plan and a focal point in relation to violence against health professionals. In addition, nurses (*p* = 0.003) and professionals who had worked at the institution for more than 7 years (*p* = 0.003) were more likely to report being aware of the existence of a focal point in their institution for violence issues.

**Figure 1:**
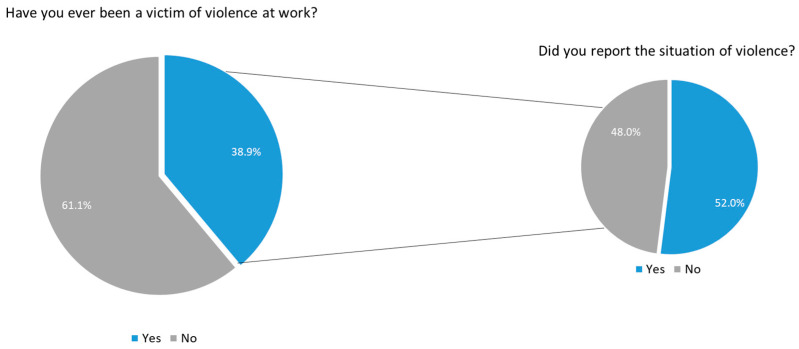
Prevalence and reporting of violence towards health professionals.

Although almost 40% of those interviewed said they had been victims of violence in the workplace, only 52% reported the incident (Figure 1). The reasons for not reporting the situation were lack of knowledge about how and where to report (17.0%), not trusting the system (12.3%), it being too complex (8.8%), undervaluing the situation (6.4%), fear of reprisals (6.3%), users being uncompensated or cognitively impaired (4.3%), the unavailability of the reporting system (2.2%), lack of time (1.1%) and being overworked (1.1%). Of those who reported violence, the majority did so through the local reporting system (41.6%), to their direct bosses (28.1%), on the National Incident Reporting System (NOTIFICA^®^) website (27.0%), to management (13.5%) and to Occupational Health services (12.4%9). Less than 6% reported to the Risk Management Support System (SAGRIS^®^) and 2.3% to the Professional Association, the Regional Health Administration, Health Event & Risk Management (HER+) and the General Inspection of Health Activities.

**Figure 2:**
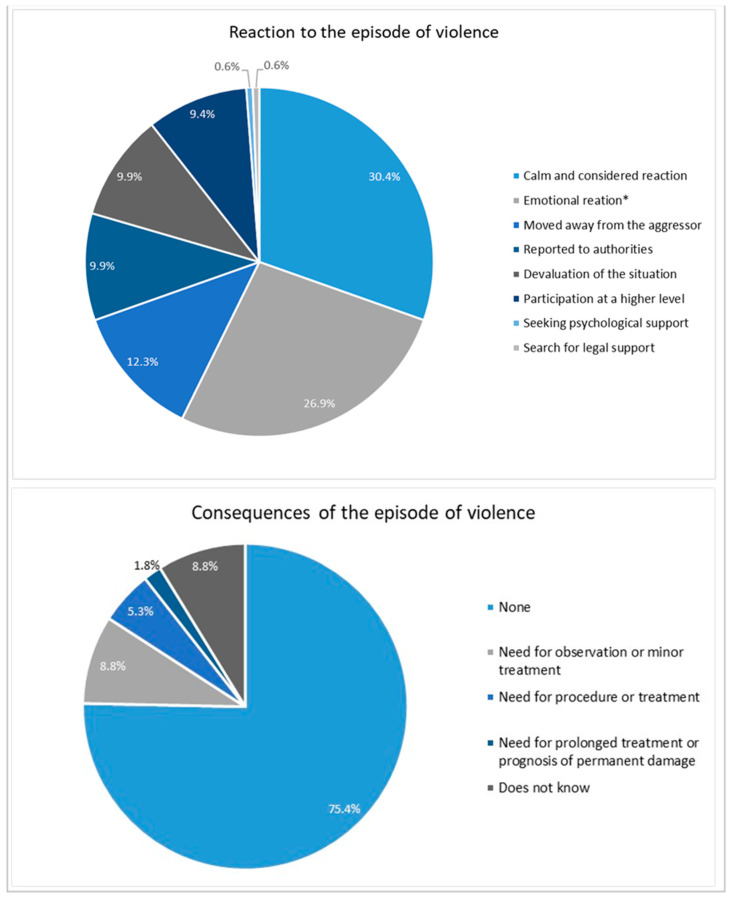
Health professionals’ reaction to and main consequences of the episode of violence. * Feelings of anger, anxiety, nervousness or fear.

Being calm and attentive (30.4%), feeling anger, anxiety, nervousness or fear (26.9%); distancing oneself from the aggressor to avoid reacting (12.3%), reporting the incident to the authorities (9.9%), devaluing the situation (9.9%) and reporting the violence experienced at a higher level (9.4%) were the most common reactions to the episode of violence (Figure 2). As for the main consequences, 75.4% of health professionals reported that there were no consequences, while 1.8% said that there was a need for prolonged treatment or a prognosis of permanent damage.

**Figure 3:**
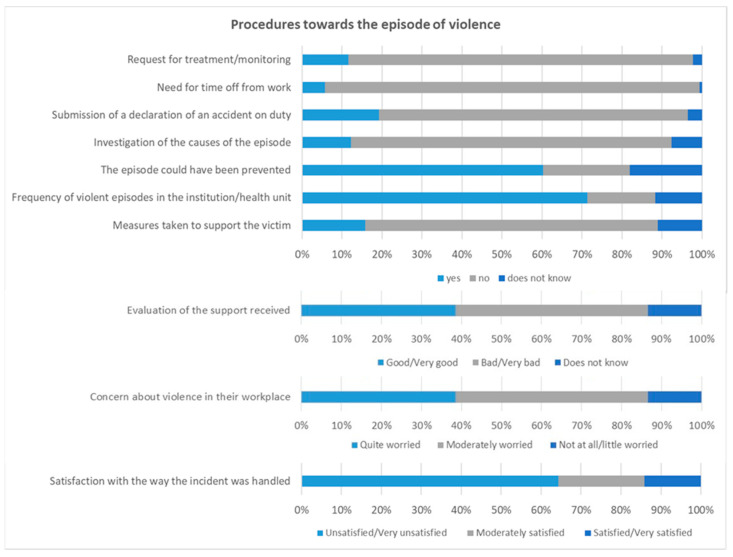
Procedures towards the episode of violence.

Figure 3 illustrates the procedures implemented at the health institution in response to the episode of violence. The majority of victims (86.0%) did not request any treatment or follow-up after the episode of violence, nor did they need to take time off work (93.6%). More than three-quarters (77.2%) did not report the accident at work to their line manager and 80.1% did not take any measures to investigate the causes of the violence. Only 12.3% of those interviewed reported that measures had been taken, namely, reporting the incident to the authorities (33.3%), internal assessment (33.3%), referring the aggressor to psychiatry (19.0%), transferring the victim from the unit where the aggression took place (10.0%) and providing psychological support to the victim (5.0%). The majority of interviewees believe that the episode of violence could have been avoided (60.2%) and that such episodes occur frequently in their institution/healthcare unit (71.3%). When evaluating the support received at institutional level, more than half rated it negatively (57.3%: 31% bad and 26.3% very bad) and said that no support measures were taken after the episode of violence (73.1%). The vast majority of respondents (86.6%) expressed concern about violence in the workplace and were dissatisfied with the way the incident was handled (64.3%: 37.4% dissatisfied and 26.9% very dissatisfied).

**Figure 4:**
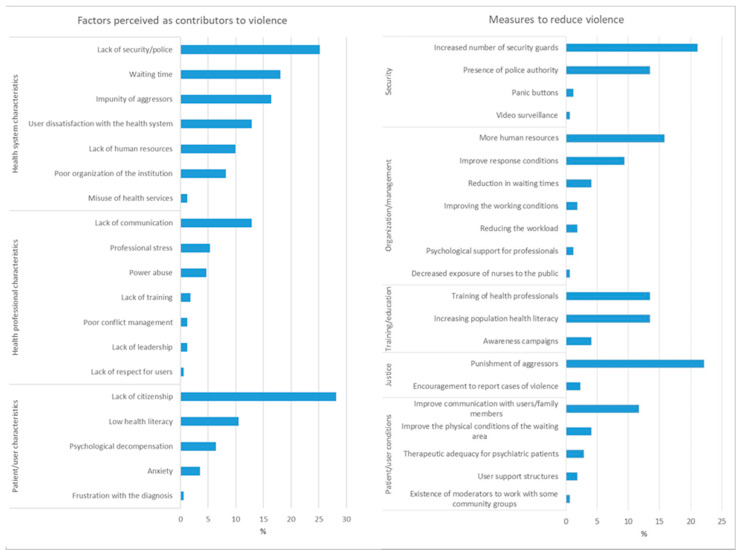
Most important factors perceived as contributors to violence against healthcare professionals and most important measures to reduce violence against healthcare professionals.

The main factors contributing to violence against health professionals can be grouped into three main themes: characteristics of the health system, characteristics of health professionals and characteristics of the user (Figure 4). The main factors associated with the health system include lack of security (26.9%), long waiting times (18.1%), impunity for the aggressor (16.4%) and user dissatisfaction with the health system (12.9%). As for health professionals, lack of communication (12.9%), professional stress (5.3%) and abuse of power (4.7%) were the most frequently reported. Factors associated with patient characteristics include lack of citizenship (28.1%), low health literacy (10.5%) and psychological decompensation in psychiatric patients (6.4%).

The interviewees identified several important measures to reduce violence against health professionals (Figure 4). The most mentioned suggestions were related to security (36.3%), especially the need to increase the number of security guards (21.1%) and the presence of police authorities (13.5%). Aspects related to the organization and management of health institutions were also mentioned (34.5%), especially the need for more human resources (15.8%), improving health response conditions (9.4%) and reducing waiting times (4.1%). Training and education were also cited, with emphasis on training health professionals in conflict management, communication and self-defense (13.5%), as well as the need to increase the population’s health literacy levels (13.5%). Measures related to justice were also mentioned (24.6%), including the need to punish aggressors (22.2%) and encouraging the reporting of cases of violence (2.3%). Finally, there were suggestions aimed at improving conditions for users (21.0%), communication with users and family members (11.7%), as well as improving the physical conditions of the waiting area (4.1%) (Figure 4).

Overall, 43.2% of health professionals reported having witnessed incidents of violence in their workplace in the 12 months prior to the survey. Most of these incidents involved male aggressors (63.2%) and female victims (77.4%), with nurses being the most affected professional group (59.5%) (Table 3). The main aggressors were service users (43.7%) and their families (28.4%), but cases involving doctors (9.5%) and nurses (8.4%) were also reported. More than three-quarters of those interviewed had witnessed psychological violence, while 17.9% had observed physical violence. However, almost 70% of participants reported that there were no consequences after the incident.

## 4. Discussion

The results of this study reveal a worrying reality regarding the phenomenon of violence against health professionals in Alentejo (southern Portugal), highlighting the lack of knowledge and low reporting of these events, limitations in reporting practices and insufficient institutional measures to deal with the consequences of this violence.

The results show that the majority of health professionals who took part in this study were female, aged between 34 and 55, and were predominantly nurses. These data reflect the typical demographic profile of the healthcare workforce, both in Portugal and internationally, in which women and nurses represent the main professional group in the healthcare sector (33% of all healthcare professionals, followed by doctors with 21%) [9,12,21,22]. Health professionals, who face a significant workload, are particularly vulnerable to violence, which further exacerbates the challenges of the work environment [12].

Although the majority of health professionals were aware of the existence of an action plan for the prevention of violence and of a focal point for reporting incidents, this study revealed a significant gap between knowledge and reporting practices. These results coincide with those found by Okubo et al. [9]. This low rate of reporting is worrying and reflects a series of barriers identified by professionals, such as a lack of knowledge about reporting procedures, distrust in the system, the complexity of the process, fear of reprisals and undervaluing the situation [9,12]. These factors indicate an urgent need to improve communication and support mechanisms within health institutions, to ensure that professionals feel safe and encouraged to report incidents of violence.

The most common reactions that health professionals expressed after an episode of violence included staying calm and being attentive, as well as feelings of anger, anxiety and fear. However, it is worrying that the majority of professionals reported that there were no significant consequences following episodes of violence and did not request treatment or psychological support, nor were they absent from work. This suggests a culture of normalizing violence in the workplace, with Nelson et al. reporting that “passive acceptance of violence against nurses persists in the legal system, perhaps because ‘patients are [considered] ill and [therefore] cannot be controlled’” [19], where professionals often feel obliged to continue their duties without adequate support. In addition, only 12.3% of respondents reported that institutional measures were taken in response to incidents, which demonstrates a serious failure in the management and prevention of future episodes of violence.

Internationally [23] and at national level [3], the importance of having effective reporting systems, supervision of leadership and of the policies and procedures of health units, as well as the collection and analysis of data on post-incident strategies, training and education to reduce violence in the workplace has been emphasized.

The factors identified by the participants as contributing most to situations of violence can be grouped into three main themes: the health system, the characteristics of health professionals and the characteristics of users. Lack of security, long waiting times and the impunity of aggressors were the most frequently cited factors related to the health system. These results underline the need for structural interventions to improve security in healthcare facilities and reduce waiting times, which could reduce user dissatisfaction and, consequently, episodes of violence. Other authors [24,25] in their research also found identical results that corroborate the findings of this study.

Participants suggested various measures to reduce violence, with an emphasis on increasing security (36.3%), including hiring more security guards and the presence of police authorities. In addition, they highlighted the need for improvements in the organization and management of health institutions, such as increasing human resources and reducing waiting times. Ongoing training for health professionals in conflict management, communication and self-defense was also pointed out as a crucial measure. These results indicate that, in order to effectively address violence against health professionals, a coordinated effort is needed, involving improvements in security, management, training and communication within health institutions, as reported in the research by Okubo et al. [9].

The results of this study have implications for health management and policies in the southern region of Portugal. There is a clear need to strengthen reporting mechanisms and support for health professionals, ensuring that reporting systems are accessible and effective [26]. Health institutions must implement clear policies and swift action in response to episodes of violence, in order to break the cycle of impunity and create a safer working environment.

In addition, it is essential to invest in the continuous training of health professionals, not only in their technical skills, but also in communication and conflict management skills [27,28]. The promotion of a culture of zero tolerance for violence and the involvement of security authorities are essential steps to protect health workers and ensure that they can perform their duties safely and with dignity [24,29].

Finally, the measures suggested by the health professionals who took part in this study should be considered by the competent authorities in order to develop a comprehensive action plan that takes into account the specific needs of health institutions in southern Portugal and that can serve as a model for other regions of the country.

### Limitations

Despite its strengths, this study has some limitations that should be mentioned. Firstly, the results may not be generalizable to other regions of Portugal with different socio-economic and cultural characteristics. Secondly, a cross-sectional study allows associations to be identified but does not establish case-match relationships. Longitudinal studies will be needed to confirm these results and explore the casual relationships between the variables.

## 5. Conclusions

Violence against health professionals in southern Portugal is an urgent issue that requires immediate attention and intervention. Increasing knowledge about this problem, improving reporting practices and understanding its consequences are fundamental steps towards creating a safer and healthier working environment. It is imperative that governments, organizations and professionals work together to implement preventive and reactive measures that protect health professionals, with the consequent improvement in the health care provided.

The adoption of effective policies and the creation of a culture of zero tolerance to violence are essential to ensure that health professionals can carry out their duties safely, benefiting both the professionals and the users of the health system.

## Figures and Tables

**Table 1:** Participants’ sociodemographic and work-related characteristics (n = 440).

Variables	n (%)
Sociodemographic characteristics	
Sex	
Female	329 (74.8)
Male	111 (25.2)
Age (years)	
23–33	63 (14.3)
34–44	161 (36.6)
45–55	119 (27.0)
56–66	78 (17.7)
>66	7 (1.6)
No answer	12 (2.8)
Nationality	
Portuguese	424 (96.4)
Other	16 (3.6)
Marital status	
Married	255 (58.0)
Cohabiting	73 (16.6)
Single	76 (17.3)
Divorced	33 (7.5)
Widowed	3 (0.6)
Work-related characteristics	
Professional category	
Nurse	352 (80.0)
Physician	88 (20.0)
Workplace	
Local Health Unit of Central Alentejo, EPE *	113 (25.7)
Local Health Unit of Alto Alentejo, EPE *	227 (51.6)
Local Health Unit of Baixo Alentejo, EPE *	27 (6.1)
Local Health Unit of Litoral Alentejano, EPE *	73 (16.6)
Type of contract	
Individual Employment Contract	147 (33.4)
Employment contract in public functions for an indefinite period	285 (64.8)
Services provision	8 (1.8)
Working time at the institution (years)	
<1	12 (2.7)
1–4	45 (10.2)
5–7	48 (10.9)
8–10	38 (8.6)
≥11	297 (67.5)
Work shifts	
Sometimes	33 (7.5)
Yes	213 (48.4)
No	194 (44.1)

* Entidades Públicas Empresariais (Public Business Entities).

**Table 2:** Knowledge on violence prevention in the health sector, according to participants’ sociodemographic and work-related characteristics (n = 440).

Variables	Action Plan			Focal Point		
	No n (%)	Yes n (%)	*p*	No n (%)	Yes n (%)	*p*
Overall	181 (41.1)	259 (58.9)		184 (41.8)	256 (58.2)	
Sex						
Female	131 (72.4)	198 (76.4)		134 (72.8)	195 (76.2)	
Male	50 (27.6)	61 (23.6)	0.333	50 (27.2)	61 (23.8)	0.425
Age (years)						
23–33	34 (19.7)	29 (11.4)		37 (20.9)	26 (10.4)	
34–54	112 (64.7)	168 (65.9)		117 (66.1)	163 (64.9)	
≥55	27 (15.6)	58 (22.7)	0.024	23 (13.0)	62 (24.7)	0.001
Nationality						
Portuguese	173 (95.6)	251 (96.9)		175 (95.1)	249 (97.3)	
Other *	8 (4.4)	8 (3.1)	0.463	9 (4.9)	7 (2.7)	0.233
Marital status						
Married/Cohabiting	126 (69.6)	202 (78.0)		125 (67.9)	203 (79.3)	
Single/Divorced/Widowed	55 (30.4)	58 (22.0)	0.047	59 (32.1)	53 (20.7)	0.007
Professional category						
Nurse	140 (77.4)	212 (81.8)		135 (73.4)	217 (84.8)	
Physician	41 (22.6)	47 (18.2)	0.245	49 (26.6)	39 (15.2)	0.003
Type of contract						
Individual Employment Contract	62 (34.3)	85 (32.8)		71 (38.6)	76 (29.7)	
Employment contract in public functions for an indefinite period	115 (63.5)	170 (65.7)		108 (58.7)	177 (69.1)	
Services provision	4 (2.2)	4 (1.5)	0.820	5 (2.7)	3 (1.2)	0.056
Working time at the institution (years)						
<5	24 (13.3)	33 (12.7)		24 (13.0)	33 (12.9)	
5–7	23 (12.7)	25 (9.7)		31 (16.9)	17 (6.6)	
≥8	134 (74.0)	201 (77.6)	0.573	129 (70.1)	206 (80.5)	0.003
Work shifts						
Yes/Sometimes	81 (44.8)	165 (63.7)		91 (49.5)	155 (60.6)	
No	100 (51.2)	94 (36.6)	<0.001	93 (50.5)	101 (39.4)	0.021

Note: * Brazilian, Cuban, Spanish and Italian.

**Table 3:** Characterization of the observed violence towards other health professionals (n = 190).

Variables	n (%)
Sex of the victim	
Female	147 (77.4)
Male	43 (22.6)
Sex of the aggressor	
Female	70 (36.8)
Male	120 (63.2)
Professional category of the victim	
Nurse	113 (59.5)
Physician	32 (16.8)
Operational assistant	14 (7.4)
Technical assistant	14 (7.4)
Security	7 (3.7)
Others	8 (4.2)
Does not know/answer	2 (1.0)
Aggressor relation with the health institution	
Patient/users	83 (43.7)
Patient/users companion	54 (28.4)
Physician	18 (9.5)
Nurse	16 (8.4)
Citizens group	3 (1.6)
Health professional from another institution/health unit	3 (1.6)
Visitors	1 (0.5)
Others	12 (6.3)
Type of violence	
Psychological	143 (75.3)
Physical	34 (17.9)
Racial/ethnic discrimination	9 (4.7)
Gender	1 (0.5)
Other	3 (1.6)
Consequences of the episode	
None	129 (67.9)
Need for observation or minor treatment	17 (8.9)
Need for procedure or treatment	9 (4.7)
Need for prolonged treatment or prognosis of permanent damage	2 (1.1)
Does not know	33 (17.4)

## Data Availability

Data are available from the corresponding author upon request.

## Public Involvement Statement

Nurses and doctors were involved. They completed a self-administered questionnaire.

## Guidelines and Standards Statement

This manuscript was drafted against the STROBE checklist for cross-sectional studies research.
